# Confronting the opioid crisis with consumer health information: a look at East Tennessee

**DOI:** 10.5195/jmla.2021.1015

**Published:** 2021-01-01

**Authors:** Kelsey Leonard Grabeel, Jenny C. Moore

**Affiliations:** 1 kgrabeel@utmck.edu, Preston Medical Library/Health Information Center, University of Tennessee Graduate School of Medicine/University of Tennessee Medical Center, Knoxville, TN; 2 jmoore2@utmck.edu, Preston Medical Library/Health Information Center, University of Tennessee Graduate School of Medicine/University of Tennessee Medical Center, Knoxville, TN

## Abstract

**Background:**

Starting in the 1990s, health care providers began prescribing opioids to patients as pain relievers, believing they were safe. However, many patients became addicted to these pills. In 2017, the US Department of Health and Human Services declared a public health emergency to fight the opioid epidemic. This crisis was prevalent in East Tennessee, where many residents were prescribed opioids.

**Case Presentation:**

Librarians at an academic medical center library in East Tennessee analyzed the health information requests related to pain, mental health, and addiction over the last fifteen years. We reviewed the pattern of requests related to these topics, the counties requesting this information, and the impact that these hospital policies had on these requests.

**Conclusions:**

From 2005 to 2014, there were few requests about mental health, pain, and substance abuse. However, once the library moved into the hospital and there was an increase in awareness of opioid addiction, requests on those topics increased. Most of the requests were about pain, with the height occurring in 2017, during which year the public health emergency to fight the epidemic was declared. Additionally, 2017 was the year the hospital implemented visitor limitations for patients with infections associated with intravenous drug use, which might explain the drastic drop in substance abuse information requests in 2018. Future outreach will target counties that have a high opioid prescription rate.

## BACKGROUND

As an important part of the health care community, it is essential for medical librarians to stay up to date on the latest health trends, pandemics, or crises in the US population. One major health crisis that health care, and therefore libraries, face is the opioid epidemic. According to the US Health and Human Services Department (HHS), health care providers began prescribing opioid pain relievers at a high rate in the late 1990s, believing they were safe [1]. Pain even became known as the fifth vital sign in order to make pain relief a part of patient well-being, which encouraged the prescription of opioids [2]. This increase in prescription rate led to widespread misuse, both for nonprescription and prescription opioids [1]. Eventually, the addictiveness of these pain relievers became apparent, and in 2016, there were 42,000 opioid overdose deaths, 40% of which involved a prescription opioid [1]. In 2017, HHS declared a public health emergency, which was called the “opioid crisis.”

In East Tennessee, use of opioids has been significant. In Knox County alone, one of the largest counties in East Tennessee and the location of the University of Tennessee Medical Center (UTMC), 347,831 opioid pain prescriptions were filled in 2018 [3]. Blount County, a county neighboring Knox, has the next highest prescription rate in the East Tennessee area, with 114,946 prescriptions [3]. In the 21-county region that UTMC serves, 1,462,663 opioid pain prescriptions were filled in 2018 [3]. The Appalachian region, which includes the area that the hospital serves, has a 65% higher mortality rate due to substance abuse than the rest of the nation, and 69% of those overdose deaths were opioid related [4].

Reducing prescription rates is one goal in combating the epidemic; however, some researchers conclude that reducing opioid prescriptions alone is no longer enough. Researchers have found that mental health also plays a role in addiction and that treatment of comorbid psychological disorders by trained mental health personnel is key to preventing overdose deaths and promoting recovery [5]. Indeed, of US adults who have a mental health condition, 18.7% use prescription opioids [6].

Poor physical and mental health are associated with increased rates of opioid prescriptions in the United States. For example, a large observational study found that poor mental health status was predictive of survey participants receiving more opioid prescriptions in the following year, sometimes up to 6 or more opioid prescriptions in the second year of the survey [7]. This may play a role in the high use of opioids in Tennessee, which ranks slightly higher than the national average for serious mental health illness in adults 18 or older [8]. Compounding mental health issues is a lack of mental health providers. An analysis of all US counties found that Tennessee has a higher ratio of population to mental health providers (660:1) compared to the national average (400:1) [9]. In a ranking to determine quality of life and life expectancy according to various health factors (including access to mental health providers), Knox, Blount, and Loudon counties scored in the top quarter among Tennessee counties, while other counties in the UTMC service area—such as Campbell, Union, and Scott—scored in the bottom quarter among all Tennessee counties, indicating that poverty, lack of education, and lack of health care are a concern in much of the region [9].

Due to the high use of opioids in East Tennessee, the Health Information Center (HIC), the consumer health division of the academic medical library located at UTMC, evaluated health information requests related to pain and substance abuse over the last fifteen years. As mental health can also have an impact on addiction, those requests were also evaluated. Our objectives were to: (1) review information requests related to these topics to identify any patterns; (2) examine which counties in the service area requested information; (3) determine whether information requests on these topics reflected the timeline and growth of the opioid epidemic in the region; and (4) identify factors that may have influenced these information requests. Based on the results of this analysis, programs and outreach in the community were implemented or explored.

## CASE PRESENTATION

### Analysis of Health Information Center requests

**Figure 1:**
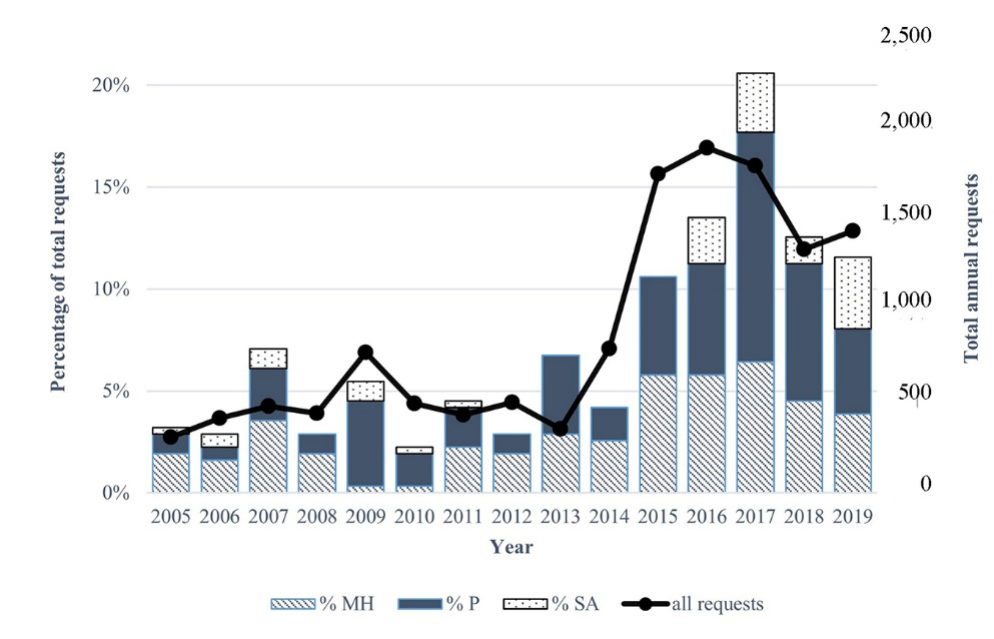
Total yearly requests about mental health (MH), pain (P), and substance abuse (SA) topics as a percentage of total yearly requests at the Health Information Center (HIC) in the Preston Medical Library in Knoxville, Tennessee

The Preston Medical Library (PML) has been providing a health information service for over twenty-five years. Since the start of the service, data have been collected on the question type and zip code of the consumer requesting the information. Up until 2014, PML was located in the University of Tennessee Graduate School of Medicine (UTGSM) building, where there were an average of thirty health information requests a month. In late 2014, PML moved inside the hospital and opened the HIC, which led to a drastic increase in health information requests (Figure 1).

The line represents total annual requests on all topics by count.

The authors examined the total yearly number of mental health, pain, and substance abuse information requests as a proportion of total requests annually in order to observe how requests on these topics changed over the past 15 years. We analyzed our internal consumer health database and received UTGSM Institutional Review Board approval (IRB # 4618). To calculate proportional data, information requests were divided into categories based on whether they referred to topics dealing with mental health, pain, or substance abuse, and the number of requests in each category were totaled for each year. Then, proportions were calculated as total requests in a category (C) divided by all total yearly requests in all categories (T), multiplied by 100 (C/T×100). It was important to examine the proportions as opposed to the numbers of information requests because requests from the public increased 217% after the move [10].

From 2005 to 2014, the proportion of pain and mental health information requests remained steady, and the proportion of substance abuse information requests was never higher than 1% of the total number of yearly information requests (Figure 1). Although there were no substance abuse information requests in 2015, the proportions of pain and mental health information requests increased in that year. Starting in 2016 and continuing through 2019, substance abuse information requests were 1.3%–2.5% of total requests, which mirrored the height of the opioid epidemic. Most notably, there was a large spike in the proportion of pain and mental health requests in 2017. Although total requests overall declined slightly in 2018 and 2019 after the 2017 peak, the proportion of information requests on mental health, pain, and substance abuse remained more than 10% of all requests (Figure 1).

### Changes in hospital policy to address the opioid epidemic

When the HHS declared a public health emergency in 2017, the hospital started making changes to their policies and protocols in regard to pain management and visitor restrictions. UTMC began a quality improvement plan aimed at reducing or eliminating opioid use for postsurgical pain treatment for one of the hospital's routine surgical procedures [11]. Furthermore, visitor restrictions for patients with intravenous drug use–associated infections were put into place [12]. Upon admission, patients were not allowed visitors, were restricted to their floors [13], and were requested to participate in addiction treatment after recovering from infection [12].

### Demographic analysis

**Figure 2:**
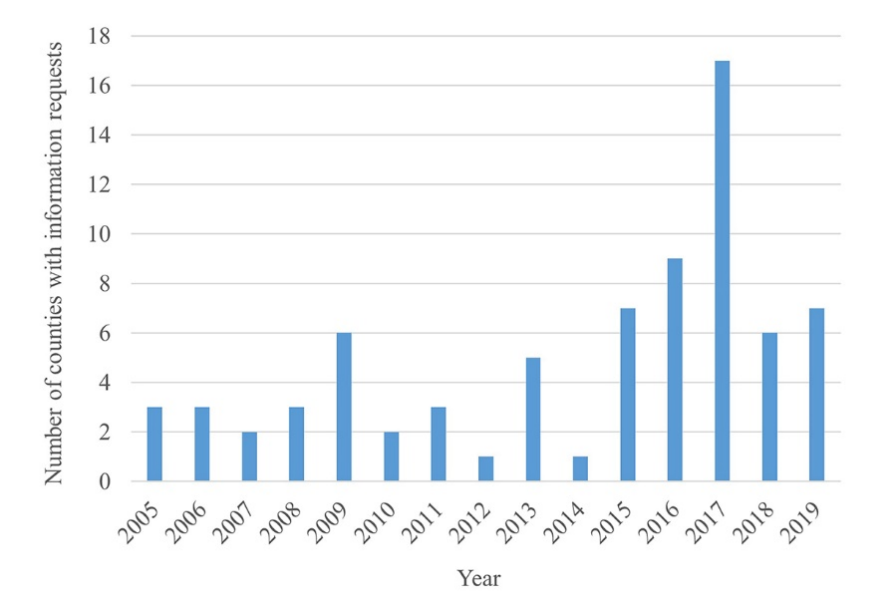
Number of different counties represented in HIC information requests about mental health, pain, and substance abuse

An analysis of the HIC information requests on the topics of mental health, pain, and substance abuse by county indicated that most requests came from Knox County (data not shown). This was expected because Knox has the largest population (third largest in Tennessee) of the twenty-one-county UTMC service area and is the county in which UTMC is located [14]. After the opening of the HIC in 2014, it appeared that a wider range of the hospital's twenty-one-county service area was reached (Figure 2).

## DISCUSSION

We found that HIC information requests mirrored the timeline of the opioid epidemic as well as the needs of our region. For example, the 2015 increase in information requests about pain and substance abuse preceded a 20% increase in total drug overdose fatalities in 2016 [15]. Additionally, the declaration of the opioid epidemic in 2017 coincided with a spike in the number of pain and mental health information requests that year. Furthermore, a 2019 UTMC community health needs analysis identified substance abuse and mental health as 2 of the top 4 needs for the service area [16], demonstrating another link between the increase in HIC information requests and the needs of the community.

There are likely many factors that influenced the number and type of information requests by patrons at the HIC. First, there was the move inside the hospital, which placed the library on the first floor of the hospital and began a shift from primarily phoned information requests to in-person, or “walk-in,” requests. Second, UTMC began to change some patient care practices, such as creating an opioid consent form for patients who were prescribed opioids that further alerted patients to the risks of using these drugs. The implementation of this consent form and increased awareness of risk might have led patients and their family members to seek more information, possibly increasing the number of information requests about pain-related topics, such as alternative pain treatments, in 2017.

Similarly, in 2018, patients with intravenous drug use–associated infections were restricted to their floors and had restrictions placed on visitation. This might explain the decrease in substance abuse questions that year, as patients could not visit the HIC and might not have learned about the health information service. Third, increased coverage of the opioid epidemic by the news media might have been involved in increasing patron requests by increasing awareness of the problem. In an analysis of news coverage of the opioid epidemic from 2007 to 2016, Kennedy-Hendricks et al. found that there was a slight uptick in coverage in 2012 before a much larger increase in 2015 and 2016 [17], which could explain why we observed increases in information requests about pain starting in 2015.

As this epidemic unfolded, we learned that patients and family members might feel uncomfortable asking about substance abuse and might instead request information about pain. Therefore, it is important for libraries to have easily accessible research guides about the opioid epidemic or books about substance abuse in case patrons feel reluctant to ask library staff for information. Based on the volume of information requests that we received, we added books on addiction, substance abuse, and pain to our consumer health collection. Although these books can be checked out, the HIC also offers comfortable seating for reading as well as free copying so that patients or family members can browse the books without informing library staff of their selection. To further bring awareness to mental health and substance abuse, an information table has been set up at the front of the library. Books on these topics are displayed as well as hand outs and a brochure advertising our health information services. *MedlinePlus* magazines are also available—specifically the summer 2019, fall 2019, and winter 2019 issues that addressed mental health and opioids [18]. The television monitor located in the HIC also displays slides on mental health and substance abuse with general facts.

We have also focused outreach on areas in the hospital that may be more populated with individuals affected by the opioid epidemic. Librarians have partnered with the neonatal intensive care unit (NICU) to bring the HIC to the family waiting room for one day every two months to provide health information and books related to the NICU, mental health, and substance abuse [19]. The space allows a table on which books, information resources, and an iPad for live searching are displayed, with a librarian available to help. It is especially important to partner with the NICU because approximately 20% of babies in the UTMC NICU have neonatal abstinence syndrome [20] and are withdrawing from opioids [21]. Librarians have also collaborated with inpatient surgery and day surgery departments to bring the HIC to their waiting rooms one day a month to provide patients and family members with information on pain management. Lastly, the Cancer Institute has also hosted the library 1 day every 3 months to provide patients and family members health information on cancer as well as pain management [19]. These outreach events are ongoing.

Furthermore, our outreach to the community that the hospital serves continues to be essential. Librarians partnered with Remote Area Medical, a pop-up clinic that provides free health care for persons without health insurance or access to care [22], to provide free health information to participants at a clinic in February 2020. At this event, participants could fill out a sheet that listed different health conditions for which they would like more information, including addiction. Information was provided to thirteen people, of whom five requested information on addiction, further reflecting the region's needs for information related to opioids and substance abuse.

Medical libraries have an important role to play in battling the opioid epidemic by providing trustworthy sources of information for patrons. In their analysis of news coverage of opioid use disorder medications, Kennedy-Hendricks et al. have found that while most news stories are accurate, some contain inaccuracies [17]. Therefore, as noted by Carnes, libraries can play a vital role in helping the communities they serve during times of crisis by providing accurate information from authoritative sources [23]. For example, the East Tennessee State University Quillen College of Medicine Library provides their community with handouts on opioid dependence at their outreach events [24]. Similarly, our Opioids and Addiction epidemic research guide can be displayed at outreach events.

Because individuals who reside in outlying counties may have limited access to computers and other resources, partnering with public libraries to provide health information services is also important, because they typically provide access to computers and are seen as community centers [25]. One way for the HIC to partner with public libraries in the UTMC service area could be to provide professional development classes for public librarians on health literacy and other topics that could help combat the opioid crisis. Real and Bogle have found that collaboration with other community and public health organizations is crucial for public libraries to deal with the opioid epidemic [26]. One successful public library program in the hospital service area is the Recovery Court at the Blount County Public Library (BCPL), which helps nonviolent drug offenders recover from addiction [27]. We have met with BCPL librarians and are planning to work with them on their opioid initiatives, including another project to educate community leaders on the opioid abuse crisis. Another area for future research would be to survey all public libraries in the twenty-one-county UTMC service area to explore how the opioid epidemic has impacted their resources and services and how they are responding.

Evaluating the HIC's information requests related to pain, substance abuse, and mental health allows us to conclude that the temporal pattern of these requests reflects the opioid epidemic timeline and demonstrates the value our library adds to the community and the hospital through its health information services. Although we cannot establish causal relationships between the hospital's policies and our information requests, we observed an overall decrease in the proportion of requests on these topics the year that visitor restrictions went into place. This analysis helps us to reflect on past and current outreach programs at the hospital and in the community and to refine our focus for future areas to expand and improve outreach. Future plans include expanding outreach at Remote Area Medical clinics, continuing our partnership with the BCPL, and exploring partnerships with other area public libraries or public health groups.

## Data Availability

Data associated with this article are available at https://dc.uthsc.edu/gsmk_facpubs/16/.
